# The genome and transcriptome of *Trichormus* sp. NMC-1: insights into adaptation to extreme environments on the Qinghai-Tibet Plateau

**DOI:** 10.1038/srep29404

**Published:** 2016-07-06

**Authors:** Qin Qiao, Yanyan Huang, Ji Qi, Mingzhi Qu, Chen Jiang, Pengcheng Lin, Renhui Li, Lirong Song, Takahiro Yonezawa, Masami Hasegawa, M. James C. Crabbe, Fan Chen, Ticao Zhang, Yang Zhong

**Affiliations:** 1 Ministry of Education Key Laboratory for Biodiversity Science and Ecological Engineering, School of Life Sciences, Fudan University, Shanghai, 200433, China; 2 School of Agriculture, Yunnan University, Kunming, 650091, China; 3 College of Chemistry and Life Sciences, Qinghai University for Nationalities, Xining, 810007, China; 4 Institute of Hydrobiology, Chinese Academy of Sciences, Wuhan, 430072, China; 5 Department of Zoology, University of Oxford, Tinbergen Building, South Parks Road, Oxford, OX1 3PS, UK; 6 Institute of Biomedical and Environmental Science & Technology, Faculty of Creative Arts, Technologies and Science, University of Bedfordshire, Park Square, Luton, LU1 3JU, UK; 7 Institute of Genetics and Developmental Biology, Chinese Academy of Sciences, Beijing, 100101, China; 8 Key Laboratory for Plant Diversity and Biogeography of East Asia, Kunming Institute of Botany, Chinese Academy of Science, Kunming, 650204, China; 9 Institute of Biodiversity Science and Geobiology, Tibet University, Lhasa, 850012, China

## Abstract

The Qinghai-Tibet Plateau (QTP) has the highest biodiversity for an extreme environment worldwide, and provides an ideal natural laboratory to study adaptive evolution. In this study, we generated a draft genome sequence of cyanobacteria *Trichormus* sp. NMC-1 in the QTP and performed whole transcriptome sequencing under low temperature to investigate the genetic mechanism by which *T*. sp. NMC-1 adapted to the specific environment. Its genome sequence was 5.9 Mb with a G+C content of 39.2% and encompassed a total of 5362 CDS. A phylogenomic tree indicated that this strain belongs to the *Trichormus* and *Anabaena* cluster. Genome comparison between *T*. sp. NMC-1 and six relatives showed that functionally unknown genes occupied a much higher proportion (28.12%) of the *T*. sp. NMC-1 genome. In addition, functions of specific, significant positively selected, expanded orthogroups, and differentially expressed genes involved in signal transduction, cell wall/membrane biogenesis, secondary metabolite biosynthesis, and energy production and conversion were analyzed to elucidate specific adaptation traits. Further analyses showed that the CheY-like genes, extracellular polysaccharide and mycosporine-like amino acids might play major roles in adaptation to harsh environments. Our findings indicate that sophisticated genetic mechanisms are involved in cyanobacterial adaptation to the extreme environment of the QTP.

The Qinghai-Tibet Plateau (QTP) is not only the highest and largest young plateau in the world, but also has the most variety of extreme environments, including rapid fluctuations in temperature, low oxygen concentration, low pressure, strong ultraviolet (UV) radiation, and severe winds. The QTP is also one of the global biodiversity hotspots with many unique environments, including snowy mountains, saline lakes, and arid deserts^1^. These environments provide an ideal natural laboratory for studies on adaptive evolution. Organisms that live in the QTP must have undergone a series of significant adaptive evolutionary genetic changes to produce a wide range of ecologically adaptive characters. Previous studies on adaptive evolution at the whole genome level on this region have focused mainly on Tibetans adapted to hypoxia (see review by Cheviron & Brumfield^2^). Recently, several genome-wide studies regarding the QTP adaptations have been conducted on non-model animals, such as yaks^3^, ground tits^4^, and Tibetan boars^5^. However, how other organisms (not animals) adapt to the QTP environments at the genomic level is still unclear.

Cyanobacteria are the earliest photosynthetic organisms; they have successfully colonized many varieties of habitats and have considerable global ecological importance^6^. Cyanobacteria can tolerate a broad range of stresses experienced in various environmental conditions, including variable osmolarity, persistent low temperatures, and high irradiance^7^,^8^,^9^. The genetic mechanisms of cyanobacterial responses to stress have been studied, especially with regard to their two-component regulatory systems, histidine and serine-threonine protein kinases, and DNA binding transcription factors^7^,^8^. Our previous field work revealed that cyanobacteria are also abundant in such extreme environments, including lakes on the QTP. The high diversity of cyanobacteria that live on the QTP indicates that they cope with harsh conditions. Among these cyanobacteria, *Trichormus* is a genus of filamentous cyanobacterium with nitrogen-fixing abilities in heterocysts. Based on akinete development, *Trichormus* was recently split from *Anabaena*^10^, which is a genus that has traditionally been used to study the genetics and physiology of cellular differentiation, pattern formation, and nitrogen fixation^11^. However, the phylogenetic relationship of these two genera was not firmly established.

To better understand how cyanobacteria evolved specific adaptations to unfavorable abiotic stress factors on the QTP, we sequenced the genome and performed deep transcriptome analysis of *T.* sp. NMC-1. This strain was isolated from Namucuo Lake, which is the largest (1920 km^2^) and highest (a.s.l. 4741 m) saltwater lake in the world. Genomic comparison between *T.* sp. NMC-1 and related species was also conducted to reveal the adaptive evolutionary pattern.

## Results

### Genome assembly and annotation

**Figure 1:**
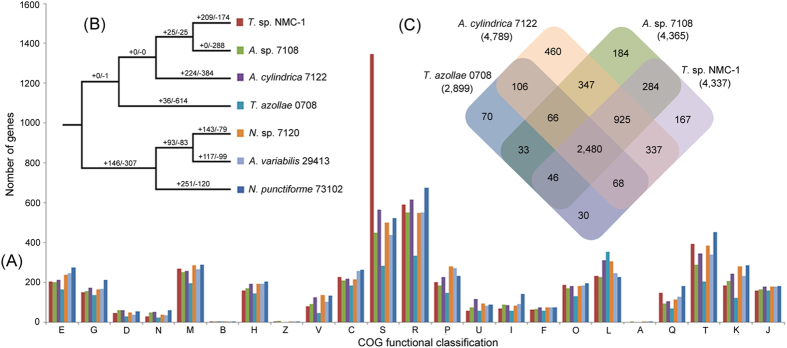
Comparative genomic analysis between *T*. sp. NMC-*1* and close relatives. (**A**) comparison of COG functional classification among the seven relatives. (**B**) The significantly (P < 0.05) expanded and contracted COG clusters in *T*. sp. NMC-1 compared with the six close relatives. (**C**) Comparison of orthogroups among the four closest relatives.

We sequenced the draft genome of *T.* sp. NMC-1 using the Illumina Genome Analyzer II platform and generated a total of 4,118,651 × 2 high qualities paired-end (PE) reads and 2,849,093 × 2 high quality mate-pair (MP) reads after the raw data were cleaned. *T.* sp. NMC-1 genome sequencing data have been deposited at NCBI BioProject under accession PRJNA324543. All sequence types provided 183-fold coverage of the genome (108- and 75-fold coverage of PE and MP data, respectively; Supplemental Table S1). After cleaned contamination, the assembly consisted of 58 scaffolds with an N50 length of 567 kb and a total genome length of 5.9 Mb (Table 1). Among these scaffolds, the 10 longest, which ranged from 1.58 to 0.17 Mb (Supplemental Table S2), covered approximately 90.5% of the assembled genome. GC content (39.2%) distributions were similar to those of other related species (Table 2). Within the genome, a total of 5,362 CDS were identified (Table 2), and 1,346 (28.12%) of these protein-encoding genes have unknown functions based on Clusters of Orthologous Groups (COG) of proteins functional categories (Fig. 1A). We then surveyed 102 housekeeping genes that were previously identified as nearly universal in bacteria^12^, and found all of these genes were present in the *T.* sp. NMC-1 draft genome. In addition, a survey of 682 core orthologous protein families from 13 cyanobacterial genomes^13^ indicated that all of these genes but *dnaA* were present in the draft genome, reported to be absent from *Synechocystis* sp. PCC 6803^14^ and *T. azollae* 0708^15^. Therefore, the sequencing and assembly results were sufficiently accurate for further comparative and evolutionary genomics analysis.

### Phylogenetic analysis based on whole genome sequences

**Figure 2:**
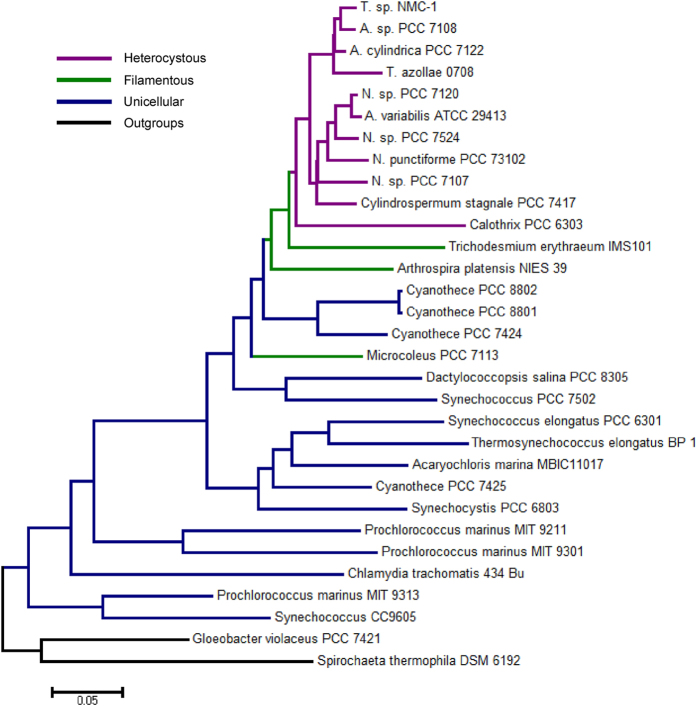
Phylogenomics of the Cyanobacteria phylum as determined using CVTree software.

Based on morphological analysis under both light and fluorescence microscopy (Supplemental Fig. S1), and 16S rRNA BLAST search in NCBI, the cyanobacterial strain from the Namucuo Lake exhibited typical morphological features of, and high sequence similarity to, *Trichormus* and *Anabaena*, which belong to the family Nostocaceae. However, because of the low rate of 16S rRNA evolution and to avoid the effect of horizontal gene transfer in cyanobacterial genomes, the CVTree without sequence alignment approach was applied to construct a phylogenetic tree. Previous research suggested that this method resolves the relationships among closely related strains better than 16s rRNA^16^,^17^. The phylogenetic tree shows that Nostocales was divided into two clusters; one cluster mainly included species from *Nostoc* and the species *A. variabilis* ATCC 29413 (Fig. 2). The strain we studied was located in the other cluster and was grouped with *A.* sp. PCC 7108, *A. cylindrical* PCC 7122, and *T. azollae* 0708 (Fig. 2).

### Evolution of COG clusters

**Figure 3:**
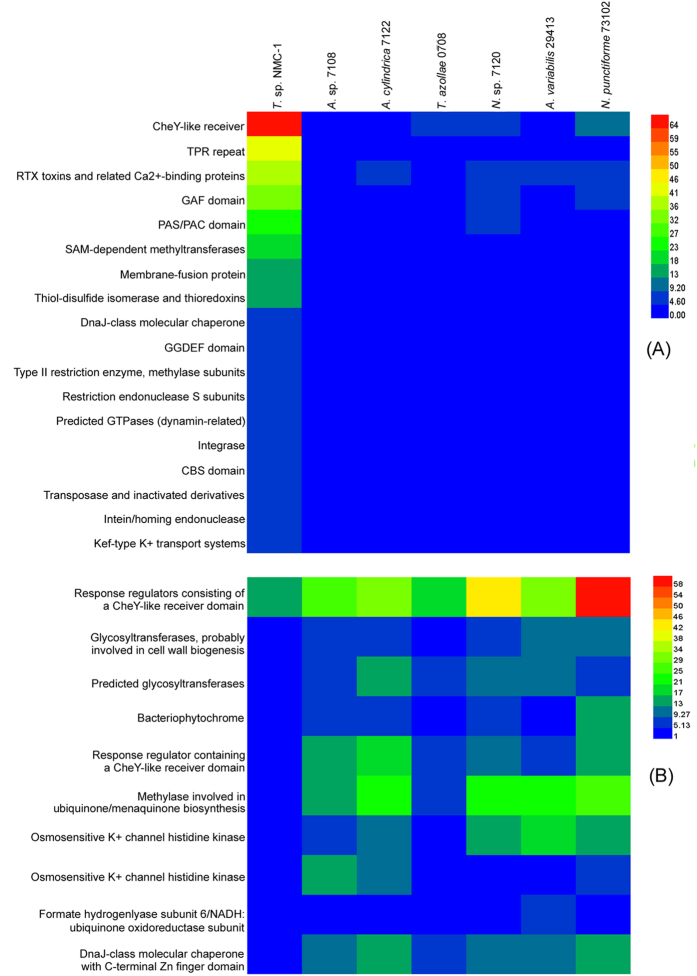
The most significantly (P < 0.0001) expanded (**A**) and contracted (**B**) COG clusters in *T*. sp. NMC-1 genome compared to six close relatives.

A total of 3295 genes were assigned to 1540 COG clusters in the *T.* sp. NMC-1 genome. Compared with the six close relatives (9.79–13.03%), an unexpectedly high proportion (28.12%) of proteins with unknown functions was detected in the *T.* sp. NMC-1 genome (Fig. 1A). Additionally, COG clusters underwent obvious expansion or contraction in the *T*. sp. NMC-1 genome. A total of 209 COG clusters substantially expanded and 174 contracted in the *T.* sp. NMC-1 genome compared with the six other genomes (Fig. 1B). In particular, 18 COG clusters were the most expanded and 10 were the most contracted (P < 0.0001, Fig. 3, Supplemental Table S4). The most significant expanded COG clusters were related to signal transduction, secondary metabolite biosynthesis, cell wall synthesis, posttranslational modification, and defense mechanisms; these clusters included COG0784 (CheY-like receiver), COG2203 (GAF domain), COG2202 (PAS/PAC domain), COG2199 (GGDEF domain, diguanylatecyclase), COG2931 (RTX toxins and related Ca^2+^-binding proteins), COG0500 (SAM-dependent methyltransferases), COG0845 (AcrA Membrane-fusion protein), COG0526 (thiol-disulfide isomerase or thioredoxin), COG2214 (DnaJ-class molecular chaperone), COG1002 (type II restriction enzyme), and COG0732 (restriction endonuclease S subunits) (Fig. 3, Supplemental Table S4).

### Identified orthogroups and genes under positive selection

A total of 5452 orthogroups (homologous gene clusters, similar to gene families) shared by *T.* sp. NMC-1 and six other related species were detected. Figure 1C shows the statistical results of the four most closely related species; there are 4170 orthogroups (including 4358 genes) in *T.* sp. NMC-1 shared with six other species, whereas 167 orthogroups (including 232 genes) are specific to *T.* sp. NMC-1. Combined with the genes (772) of *T.* sp. NMC-1 not clustered in orthogroups, there are 1004 genes specific to *T.* sp. NMC-1. Of these specific genes, 236 genes have known COG functions and are involved in adaptation, such as cell wall/membrane biogenesis, and defense mechanisms (Supplemental Table S3).

**Figure 4:**
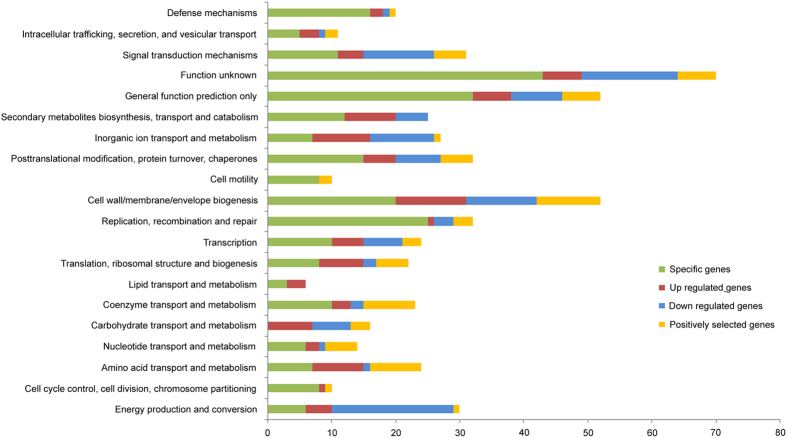
COG enrichment analysis of species-specific genes, differentially expressed genes and positively selected genes in *T.* sp. NMC-1.

In 5452 gene clusters, 2204 single-copy number (one-to-one) orthologs were shared by all seven species. For these single-copy number orthologs, the branch-site model of the PAML 4 package^18^ was used to detect genes with signals of positive selection. Finally, 491 possible genes under positive selection were identified in the *T.* sp. NMC-1 genome (ω > 1); of these genes, 70 showed highly significant evidence of positive selection (P < 0.01) (Supplementary Table S5). These 70 positively selected genes were also enriched in functions related to adaptation, such as amino acid/nucleotide/carbohydrate/coenzyme transport and metabolism (26 genes), cell wall/membrane biogenesis (10 genes), signal transduction (five genes), and posttranslational modification, protein turnover, and chaperones (five genes), by functional annotation (Fig. 4).

### Transcriptome sequencing and analyses

Global gene expression profiles of *T.* sp. NMC-1 under cold conditions were examined using transcriptome sequencing (Table 3). Finally, we generated 31.1–40.35 million clean reads and 3.12–4.04 Gb of RNA-seq data in treated and control strains after quality filtering (Table 3). The clean data were submitted to the NCBI Sequence Reads Archive (SRA) database (no. SRR3597124). All of the Pearson correlations between biological replicates were greater than 0.95, which indicates high reliability of the experiment and rationality of sample selection (Supplemental Fig. S2). The transcriptome data of control and treatment were mapped to our *T.* sp. NMC-1 genome assembly and yielded 5,362 predicted protein-coding genes and 1,023 novel transcripts. Compared with the control strain, the cold-treated strain had 312 genes with significantly altered expression after 3 d (FDR < = 0.001). COG and KEGG enrichment analyses were carried out for the up- and down-regulated genes, respectively (Supplemental Table S6). According to the COG categories, significantly up-regulated genes included those involved in processes such as membrane biogenesis, translation, ribosomal structure and biogenesis, secondary metabolites biosynthesis, amino acid transport and metabolism, and defense mechanisms (Fig. 4; Supplemental Table S6). In contrast, down-regulated genes were primarily involved in processes such as signal transduction mechanisms, membrane biogenesis, and energy production and conversion; however, some down-regulated genes had unknown functions (Fig. 4; Supplemental Table S6).

## Discussion

In this study, an alignment-free method, CVTree, was used to construct a phylogeny based on 31 cyanobacteria whole genomes (Fig. 2). The topology of our phylogenetic tree is consistent with morphological classification (unicellular, filamentous and heterocystous cyanobacteria) as well as previous studies that analyzed 16S rRNA and dozens of conserved proteins^10^,^13^,^19^. *Trichormus* sp. NMC-1 was most closely related to and had a similar genome size and amount of gene content as *A.* sp. PCC 7108. In our phylogenetic tree, it is difficult to distinguish *Trichormus*, *Anabaena*, and *Nostoc*s trains, which is consistent with a previous phylogenetic study^10^. The results indicate that these three genera are phylogenetically heterogeneous and genetically inconsistent with the morphological taxonomy. It is notable that there were similar G+C contents within each of two distinct clusters (*Trichormus* & *Anabaena* vs. *Nostoc* & *Anabaena*) (Table 2). This similarity in the same clusters indicates that G+C content could be used as a potential character for taxonomic identification in Nostocaceae.

The *T.* sp. NMC-1 genome possessed a very high proportion of genes with unknown functions compared with the other six related species based on COG category comparison. This result indicates that the *T.* sp. NMC-1 genome might have rapidly evolved after diverging from a common ancestor of *T.* sp. NMC-1 and *A.* sp. PCC 7108 to adapt to the extreme conditions of the QTP. Except for the genes with unknown functions, the obviously expanded genes in *T.* sp. NMC-1 were involved in signal transduction pathways (e.g., CheY-like receiver and related genes), secondary metabolites biosynthesis (e.g., SAM-dependent methyltransferases), cell wall/membrane biogenesis (e.g., membrane-fusion proteins), and energy production and conversion (e.g., thiol-disulfide isomerase and thioredoxins) (Supplemental Table S6). Similarly, some genes involved in the above mentioned pathways also significantly contracted. It has been reported that significant changes of gene number in one gene family was related to a major mechanism underlying the adaptive divergence of closely related species^20^,^21^. Therefore, dramatic fluctuation of these categories of gene families might reflect adaptation of the *T.* sp. NMC-1 to the extreme conditions of the QTP.

Orthologs are homologous genes that have evolved from one ancestral gene by speciation, and orthologs that show positive selection have usually undergone adaptive divergence^22^. Our results revealed 70 genes that underwent significant positive selection in the *T.* sp. NMC-1 genome based on a branch-site model. Most of these genes were related to specific adaptation traits, such as cold resistance (10 genes related to cell wall/membrane biogenesis), signal transduction mechanisms (CheA signal transduction histidine kinase), energy metabolism (fructose-1,6-bisphosphatase, alpha-mannosidase, and Fe-S-cluster-containinghydrogenase), and UV radiation resistance (caffeoyl-CoA O-methyltransferase). Interestingly, these enriched gene functions are similar to those of specific genes, and significantly expanded and contracted orthologs. Therefore, all of these consistent results indicate that *T.* sp. NMC-1 evolved complex strategies for adapting to the extreme environments in the QTP. In the following paragraphs, we will discuss the relationship between functions of these genes and the adaptation of *T.* sp. NMC-1 on the QTP.

Cyanobacteria that live in the QTP must sense and respond to various external stimulus signals from the harsh environment. Resistance to all of these stimuli should first start at signal transduction. The histidine kinase two-component systems are conserved as potential candidates of sensors and transducers of environmental signals in cyanobacteria^7^,^9^. The histidine kinase CheA and its substrate, the response regulator CheY, are partners in the two-component signaling pathway. It is noteworthy that there were five genes involved in signaling that showed significant positive selection in the *T.* sp. NMC-1 genome; of these, two genes contained the CheY-like receiver domain. The positive selection could induce gene functional changes under natural environmental stress. These data are consistent with previous studies, which suggested that CheA and CheY proteins, both contain Che receiver domain, are involved in response and adaptation to external stimuli^23^. In addition to positive selection, transcriptional changes of CheY-like receiver genes also highlighted the importance of CheY-like receiver genes contributing to cold stimuli adaptation. As the transcriptome sequencing results show that five CheY-like receiver and related regulator genes had significantly up-regulated and down-regulated expression during cold treatment (Supplementary Table S6). Furthermore, the COG0784 (CheY-like receiver) was the most expanded COG cluster in *T.* sp. NMC-1, and COG0745 (response regulators that include a CheY-like receiver domain) was the most significantly contracted COG cluster compared with its close relatives. The dramatic fluctuation of CheY gene families in *T.* sp. NMC-1 suggests adaptive divergence of closely related species. Based on the above analysis, positively selected, and differentially expressed CheY-like receiver genes with drastic, frequent turnover in *T.* sp. NMC-1 further corroborates that these genes are involved in response to cold stress, and correlated with adaptation to the harsh conditions of the QTP.

Organisms that live on the QTP must face a number of growth-related challenges from the rapid temperature changes, including decreased rates of enzyme activity, reduced fluidity of lipid membranes, and enhanced stability of nucleic acids^24^,^25^. *T*. sp. NMC-1 cells grow as aggregates and are often surrounded by a mucilaginous sheath, which is composed of components such as extracellular polysaccharide (EPS), cell surface-associated proteins, and pigments. Based on the transcriptome of *T.* sp. NMC-1 exposed to low temperatures, the category of cell wall/membrane biogenesis had the most up-regulated and major down-regulated genes, including those related to glycosyltransferases, the δ-70-transcription factor, and a membrane-bound lytic mureintransglycosylase (Fig. 4), which are involved in EPS biosynthesis^26^. In addition, out of the 70 genes that showed positive selection in the *T.* sp. NMC-1 genome, nine were involved in cell wall/membrane biogenesis and included five glycosyltransferase genes (COG0438) and one δ-70 transcriptional factor. These results indicated that EPS is important for cold adaptation of *T*. sp. NMC-1, unfavorable environmental conditions such as temperature, UV radiation, or osmotic pressure^8^,^27^. Similarly, EPS and the cell wall metabolism protein family were previously shown to be expanded for cold adaptation in the genome of *Coccomyxa subellipsoidea*, which is a polar unicellular micro alga^28^. Apart from EPS, we also identified four up-regulated genes involved in lipid transport and metabolism under cold treatment, two of which are fatty acid desaturases, namely desA and desB (>2.5-fold, Supplementary Table S6). Moreover, one RNA-binding protein (RbpB) was also induced, which has been reported to maintain *desA* and *desB* mRNA levels^29^. These expression changes could increase the proportion of unsaturated fatty acids with decreasing temperature. Therefore, these results indicate that genes related to aspects of the cell wall/membrane (e.g., EPS and membrane lipid biogenesis) in *T.* sp. NMC-1 play major roles in response to cold conditions.

**Figure 5:**
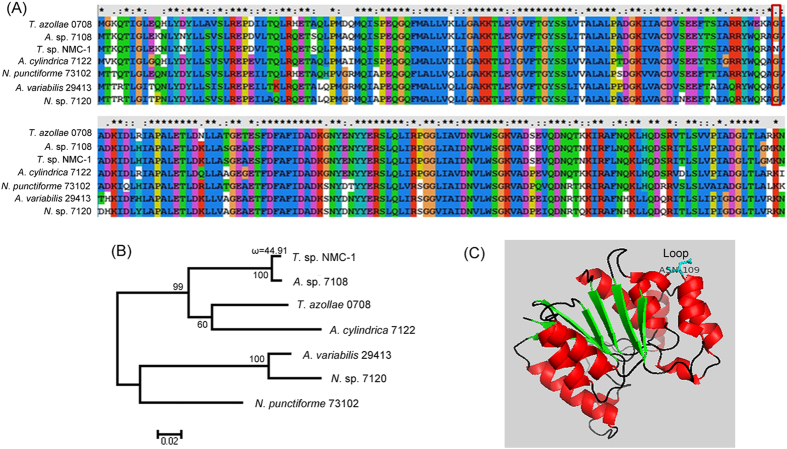
Positive selection analysis of O-methyltransferase in *T*. sp. NMC-1. (**A**) Multiple sequence alignment of O-methyltransferase. (**B**) Positive selection test of seven relatives using the branch-site model in the PAML 4 package. (**C**) Predicted three-dimensional structure of O-methyltransferase in *T*. sp. NMC-1. The positive-selection site (Asn-109) is labeled.

The QTP has the strongest UV-B radiation during the summer in the world^30^. The highly energetic UV radiation is harmful to all organisms, because it damages DNA and proteins. Marine organisms, including some cyanobacteria, have evolved to prevent UV-induced damage by synthesizing UV-absorbing/screening compounds such as mycosporine-like amino acids (MAAs)^31^,^32^,^33^. MAAs belong to a family of more than 20 compounds that absorb UV radiation. Some species of cyanobacteria have the ability to biosynthesize MAAs, whereas others lack this ability^34^,^35^. Of six closely related species, only *T. variabilis* ATCC 29413 was able to synthesize MAAs^34^. A short four-enzyme pathway of MAA biosynthesis was identified and included dehydroquinate synthase (DHQS), O-methyltransferase (O-MT), ATP-grasp, and nonribosomal peptide synthetase (NRPS) homologs^36^. BLAST searches of both DNA and protein sequences in *T.* sp. NMC-1 revealed that three genes (*dhqs*, *o-mt*, and *nrps*) and several other genes that contain highly similar ATP-grasp domains were identified. Furthermore, complex MAA compositions that included shinorine, palythine-serine, asterina330, and palythenic acid were identified in *T.* sp. NMC-1using HPLC-ESI-MS/MS methods (Supplemental Table S8, Fig. S3). It is notable that the gene encoding O-MT showed significant positive selection in *T*. sp. NMC-1 compared with related species. Additionally, an amino acid residue at position 109 in the loop of O-MT three-dimensional structure, asparagine (N), was detected under significant positive selection in *T*. sp. NMC-1; the typical amino acid residue at this site is glycine (G) in other related species (Fig. 5). Loops often play an important role in a protein’s three-dimensional structure and act as the active site of an enzyme or binding site of a receptor. Therefore, we speculate that *o*-*mt* in *T*. sp. NMC-1 underwent positive selection during adaptation to the QTP environment, and this site may have a specific function. In fact, in addition to their role as sunscreen compounds, MAAs are also involved in antioxidant, osmotic stress, and desiccation resistance^37^,^38^,^39^. Therefore, MAAs might play multiple roles in the adaptation of *T*. sp. NMC-1 to the various extreme environments of Namucuo Lake, which has high radiation exposure and salinity.

## Conclusion

Interpretation of genetic variation at the whole genome level can contribute to understanding how organisms adapt to changing environments. Organisms that live in the QTP must have undergone a series of significant adaptive evolutionary changes to produce a wide range of ecologically adaptive characters. In this study, we sequenced the draft genome and whole transcriptome of *T.* sp. NMC-1 strain in Tibet and conducted evolutionary analyses based on comparative genomics. Our findings show that positively selected and enhanced genes were involved in signal transduction mechanisms, cell wall/membrane biogenesis, secondary metabolite biosynthesis, defense mechanisms, and energy production and conversion, all of which relate to the specific adaptation traits found in this challenging environment. In particular, we found that the CheY-like genes, extracellular polysaccharide and mycosporine-like amino acids may play major roles in responding to external harsh environments. Our findings indicate that sophisticated genetic mechanisms are involved in *T*. sp. NMC-1 adaptation to the extreme environment of the QTP.

## Material and Methods

### Isolation, culture and identification of strains

The original collection of *Trichormus* sp. strain (NMC-1) was conducted on June 15, 2011 in the Namucuo Lake (N30^o^ 46.45’, E90^o^ 52.01’) in the QTP of the South West of China. The lake water was stored at 4 °C at night and then transferred to the School of Life Sciences, Fudan University in Shanghai by air the next day. The *T.* sp. NMC-1 was isolated using previously described micropipette washing methods^40^. Then each single trichome looking like *Trichormus* under the microscope was transferred to 250 ml sterilized glass Erlenmeyers containing 50 ml of MA medium^41^ and maintained at 28^o^C/23 °C with a 16/8 h (light/dark) diurnal cycle (light intensity 2200lux). High quality genomic DNA was extracted from the sample using the Genomic DNA Kit (Tiangen Biotech Co., China) following the manufacturer’s instructions. The *T.* sp. NMC-1 purification procedure was validated by PCR using cyanobacterial 16S rRNA gene specific primers^42^.

### DNA library construction, genome sequencing, assembly and annotation

The 300 bp paired-end (PE) and 3500 bp mate-pair (MP) DNA libraries were sequenced on the Illumina Genome Analyzer II system. Library preparation, sequencing and base calling were performed according to the manufacturer’s user guide (Illumina, Inc). The raw sequence reads with adapter contamination, PCR duplicates, and low-quality sequences (Q < 20) were cleaned from the initial sequencing output using custom scripts.

The genome sequence of the *T.* sp. NMC-1 was assembled using SOAP *de novo*^43^, which employs the *de Bruijn* graph algorithm in order to reduce computational complexity. We first assembled the reads from the short insert size of 300 bp into contigs using Kmer (31-mers) overlap information, and then used the mate-pair libraries, step by step from the shortest to the longest insert size, to join the contigs into scaffolds. We also cleaned contamination scaffolds from other bacteria based on a combination of protein annotation, percent GC nucleotide composition and assembly coverage depth.

ORFs and amino acid sequences were predicted from all scaffolds using the gene finding program GeneMark^44^ and Glimmer3^45^. Functional annotation of CDSs was performed through Blastp searches against GenBank’s non-redundant (nr) protein database and UniProtKB/Swiss-Prot protein database^46^. COG (Clusters of Orthologous Groups of proteins)^47^ functional categories were assigned to CDS according to DOE-JGI Standard operating procedures^48^. These data sources were combined to assert a product description for each predicted protein.

### Phylogenetic tree based on whole genome sequences

The phylogenetic tree was produced based on whole genome sequences with an alignment-free and parameter-free phylogenetic tool, CVTree ver. 2.0^49^. This method circumvents the ambiguity of choosing the genes for phylogenetic reconstruction and avoids the necessity of aligning sequences of essentially different length and gene content^17^. In total, 31 sequenced cyanobacterial genomes were chosen to produce the phylogenetic tree, using *Gloeobacter violaceus* PCC 7421 and *Spirochaeta thermophila* DSM 6192 as outgroups.

### Identification of COG clusters and orthologs between *T*. sp. NMC-1 and close relatives

Based on the phylogenetic tree, we selected genomes of six close relatives (*T. azollae* 0708*, T. variabilis* ATCC 29413, *A.* sp. PCC 7108*, A. cylindrical* PCC 7122*, Nostoc.* sp. 7120, and *N. punctiforme* PCC 73102) and *T.* sp. NMC-1 to identify COG clusters and orthologs. To identify COG clusters that had undergone expansion or contraction along each branch of the phylogenetic tree, the software package Café^50^ was applied, which is based on a likelihood model, to infer the change in gene family size. The COG clusters of the *T.* sp. NMC-1 were compared with the other six genomes, and significant levels of expansion and contraction were determined at 0.05 with lambda value equal 12 based on node numbers in the phylogenetic tree.

Furthermore, to define a set of conserved genes for cross-taxa comparison, we used OrthoMCL software^51^ to identify orthologous gene clusters (orthogroups) among the seven genomes. OrthoMCL was run with an e-value cut-off of 1e-5 and an inflation parameter of 1.5. Genes that were not included in any orthogroups, or only present in one species of orthogroups, were defined as species specific genes. The set of genes recovered from this procedure are listed in Supplementary Table S3. A Venn diagram of shared or specific gene families in *T*. sp. NMC-1 and the closest relatives (*T. azollae* 0708*, A.* sp. 7108, and *A. cylindrical* 7122; for convenience, we omitted the resource center name) according to our phylogenetic results (Fig. 1C) was conducted using R 2.2.1.

### Positive selection analysis

Positive selection can be inferred from a higher proportion of nonsynonymous (Ka) over synonymous substitution (Ks) per site (Ka/Ks > 1). In this analysis, only single-copy genes that were shared by all seven genomes were considered. To calculate the nonsynonymous and synonymous substitution rates for each one-to-one ortholog, alignment at amino acid level for each orthogroup was generated in MUSCLE^52^ using default settings. Then the resulting protein alignments were reverse-translated to codon-based nucleotide alignments with PAL2NAL^53^. For each alignment, a gene tree was constructed by RAxML software^54^ using GTR+GAMMA model, the maximum likelihood criteria. Using each gene tree topology, we applied the improved branch-site model^55^ implemented in codeml from PAML 4 package^18^ to estimate the Ka/Ks substitution rates (ω value) for each orthogroup respectively. A foreground branch was specified as the clade of *T.* sp. NMC-1. A significant likelihood ratio test (LRT) was conducted to determine whether positive selection is operating in the foreground branch. In this study, the highly significant positively selected genes were inferred only if the P-value was less than 0.01. We also detected positively selected sites if their posterior probability was greater than 95% based on empirical Bayes analysis^56^.

### Transcriptome sequencing and analyses

*T.* sp. NMC-1 cells were grown to the mid-logarithmic phase before cold stress treatment. Then the strains were cultured at 10 °C (treatment) for six hours each day within three days, and these experiments were performed as three biological replicates. Low temperature (10 °C) was selected to reflect the *in situ* low temperature (according to our investigation) in the Namucuo Lake. Two samples were cultured at 28 °C as the control group. After treatment, total RNA was extracted from *T.* sp. NMC-1 samples separately. The quality of the RNA samples was examined using the Agilent 2100 Bioanalyzer. Library construction and Illumina sequencing was performed at Novogene Bioinformatics Technology Co., Ltd (Beijing, China). An RNA-seq analysis was performed according to the protocol recommended by the manufacturer (Illumina Inc.). The reads from different conditions were mapped to the whole-genome assembly using Bowtie 2-2.0.6^57^. HTSeq 0.6.1^58^ was used to count the read numbers mapped to each gene (Table 3). And then RPKM (reads per kilobase per million reads) of each gene was calculated based on the length of the gene and reads count mapped to this gene.

Differential expression analysis of two conditions (control and cold treatment with three biological replicates) was performed using the DESeq R package (1.10.1). Genes with an adjusted P-value < 0.05 found by DESeq were assigned as differentially expressed. COG classification and Pfam domain assignment were conducted on the different expression genes (DEG). The KOBAS software^59^ was used to test the statistical enrichment of differential expression genes in KEGG pathways.

## Additional Information

**How to cite this article**: Qiao, Q. *et al*. The genome and transcriptome of *Trichormus* sp. NMC-1: insights into adaptation to extreme environments on the Qinghai-Tibet Plateau. *Sci. Rep.* **6**, 29404; doi: 10.1038/srep29404 (2016).

## Supplementary Material

Supplementary Dataset 1

Supplementary Dataset 2

Supplementary Dataset 3

Supplementary Dataset 4

Supplementary Dataset 5

Supplementary Dataset 6

Supplementary Dataset 7

Supplementary Dataset 8

Supplementary Figure S1

Supplementary Figure S2

Supplementary Figure S3

## Figures and Tables

**Table 1:** Statistics of assemble data in genome sequencing.

Statistics of contigs data		Statistics of scaffolds data	
No. of all contigs	123	No. of all scaffolds	58
Bases in all contigs	5,897,265 bp	Bases in all scaffol ds	5,938,148 bp
No. of large contigs(>1000 bp)	92	No. of large scaffords (>1000 bp)	38
Bases in large contigs	5,876,594 bp	Bases in large scaffolds	5,922,589 bp
Largest length of contigs	317,061 bp	Largest length of scaffords	1,580,590 bp
N50 length of contigs	156,762 bp	N50 length of scaffords	566,878 bp
N90 length of contigs	50,741 bp	N90 length of scaffords	169,940 bp
		N rate	0.688%
		G+C content	39.18%
		No. of CDSs	5362

**Table 2:** Genome structure of *T.* sp. NMC-1 and six close relatives.

Genome Features	Length (Mb)	G+C content (%)	Total ORF	Homologs	rRNA	tRNA
*T*. sp. NMC-1	5.94	39.18	5362	4590	12	45
*T. azollae* 0708	5.49	38.3	5380	3093	12	44
*A*. sp. PCC 7108	5.89	38.78	5169	4571	12	43
*A. cylindrica* PCC 7122	7.06	38.79	6182	5187	12	61
*N.* sp. 7120	7.21	41.2	6213	4852	12	48
*N. punctiforme* 73102	9.06	41.3	7164	4935	12	88
*T. variabilis* ATCC 29413	7.11	41.4	5813	4762	12	47

**Table 3:** Number and length of reads and number of expressed genes detected by RNA sequencing in control and cold treated samples of *T*. sp. NMC-1.

Sample name	Total clean reads	Clean bases (G)	Q20 (%)	Q30 (%)	Num. of expressed genes	Num. of highly expressed genes (RPKM > 60)
Control_1	34444484	3.54	97.62	92.01	5091	2323
Control_2	35690982	3.56	97.72	92.22	5118	2499
Cold_1	31104996	3.12	97.52	91.57	5115	2558
Cold_2	40350844	4.04	97.51	91.59	5151	2698
Cold_3	32592572	3.26	97.58	91.81	5142	2708
