# Supplementary material for: The genome and transcriptome of Trichormus sp. NMC-1: insights into adaptation to extreme environments on the Qinghai-Tibet Plateau
Source: Sci Rep. 2016 Jul 6;6:29404. doi: 10.1038/srep29404 (PMC4933973; doi:10.1038/srep29404)
Supplement: Supplementary Figure S1 [file srep29404-s9.pdf]

The genome and transcriptome of *Trichormu s*p. NMC-1: insights into adaptation to extreme environments on the Qinghai-Tibet Plateau

Qin Qiao[1, 2*], Yanyan Huang[1*], Ji Qi[1], Mingzhi Qu[1], Chen Jiang[1], Pengcheng Lin[3], Renhui Li[4], Lirong Song[4], Takahiro Yonezawa[1], Masami Hasegawa[1], M. James C. Crabbe[5,6], Fan Chen[7], Ticao Zhang[8], Yang Zhong[9, 1]

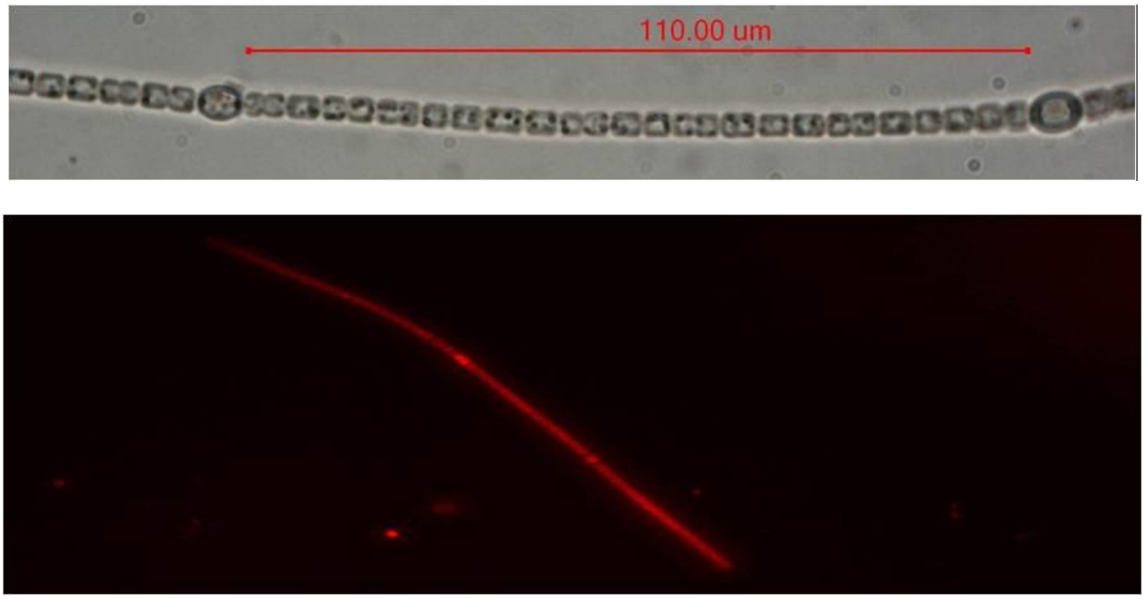

Fig. S1 Micrograph of *T*. sp. NMC-1 under light and fluorescence microscopy.
