# Supplementary material for: The genome and transcriptome of Trichormus sp. NMC-1: insights into adaptation to extreme environments on the Qinghai-Tibet Plateau
Source: Sci Rep. 2016 Jul 6;6:29404. doi: 10.1038/srep29404 (PMC4933973; doi:10.1038/srep29404)
Supplement: Supplementary Figure S3 [file srep29404-s11.pdf]

The genome and transcriptome of *Trichormu s*p. NMC-1: insights into adaptation to extreme environments on the Qinghai-Tibet Plateau

Qin Qiao[1, 2*], Yanyan Huang[1*], Ji Qi[1], Mingzhi Qu[1], Chen Jiang[1], Pengcheng Lin[3], Renhui Li[4], Lirong Song[4], Takahiro Yonezawa[1], Masami Hasegawa[1], M. James C. Crabbe[5,6], Fan Chen[7], Ticao Zhang[8], Yang Zhong[9, 1]

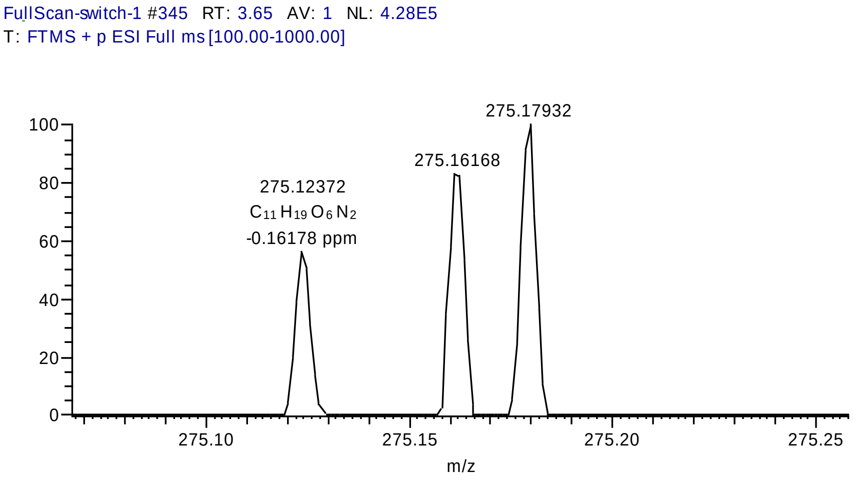

Palythine-serine:

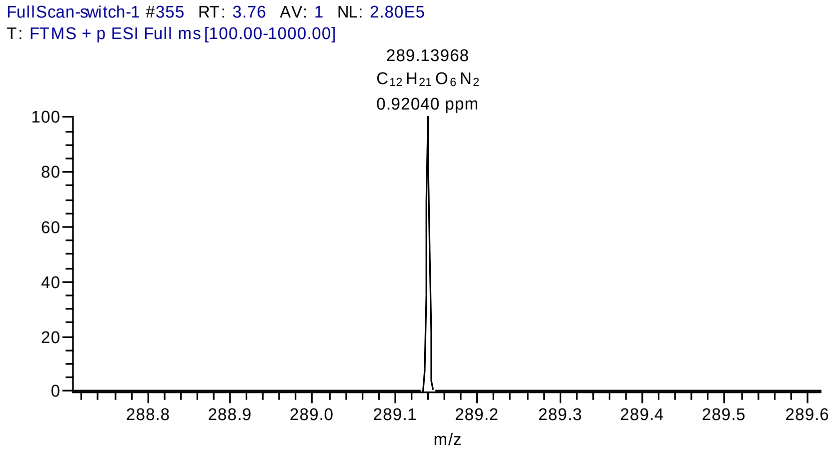

asterina330:

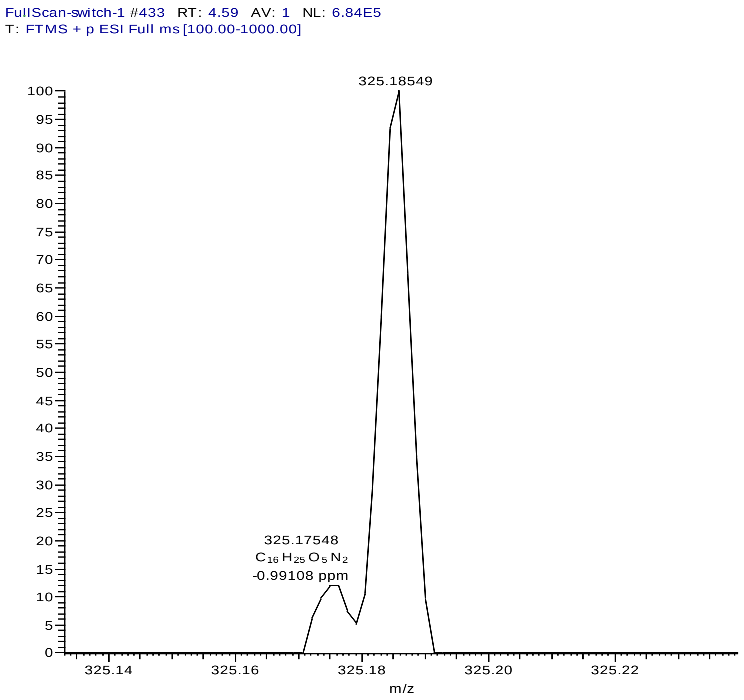

Palythenic acid:

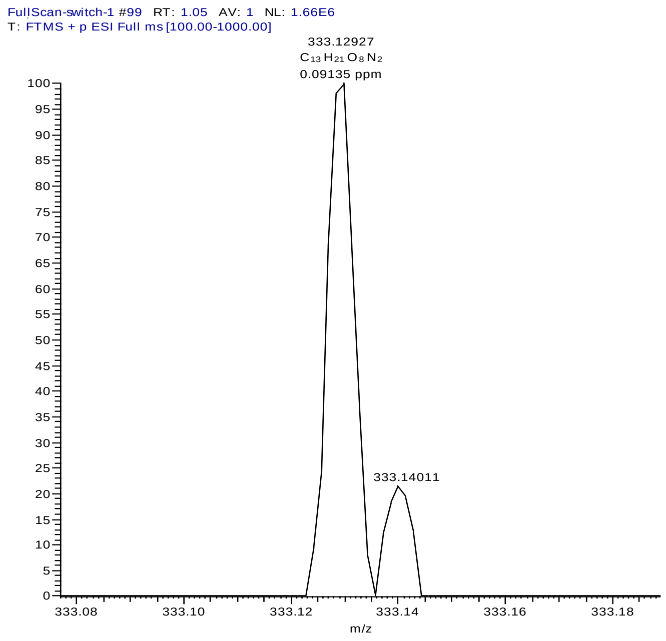

Shinorine:

Fig. S3 The primary mass spectrum of four MAAs composition in *T.* sp. NMC-1 using HPLC-ESI-MS/MS methods. HPLC parameters: chromatographic column: Sycronics C18 (100×2.1mm, 3μm); column temperature: 30 °C; mobile phase: 0.1% Formic acid /methanol; flow rate：0.3 mL/min; UV parameters: Four-channel detection, 254 nm (UV_VIS_1), 290 nm (UV_VIS_2)，310 nm (UV_VIS_3)，330 nm (UV_VIS_4); PDA scanning range: 220-450 nm. MS parameters: HESI-II, Spray voltage: 3.2KV(+)/3.0KV(-); Sheath Gas Pressure: 35 arb; Aux Gas Pressure: 10 arb; Capillary Temp: 350°C; Heater Temp: 320 °C; Scan mode: Full MS (Resolution 70,000) and dd-MS^2 (Resolution 17,500, NCE30, Stepped NCE50%); Scan range: *m/z* 100~1000.
